# Reestablishment of *Notopygos megalops* McIntosh, description of *N. caribea* sp. n. from the Greater Caribbean and barcoding of “amphiamerican” *Notopygos* species (Annelida, Amphinomidae)

**DOI:** 10.3897/zookeys.223.3561

**Published:** 2012-09-28

**Authors:** Beatriz Yáñez-Rivera, Luis Fernando Carrera-Parra

**Affiliations:** 1 Posgrado en Ciencias del Mar y Limnología, Universidad Nacional Autónoma de México, Unidad Académica Mazatlán, Joel Montes Camarena s/n, Ap. Postal 811, 82000, Mazatlán, Sinaloa, Mexico; 2 El Colegio de la Frontera Sur, Unidad Chetumal, Depto. Ecología Acuática, Av. Centenario km 5.5, 77014, Chetumal, Quintana Roo, Mexico

**Keywords:** Amphiamerican, DNA barcoding, pigmentation pattern, taxonomy, polychaete

## Abstract

The species of the genus *Notopygos* Grube, 1855 are characterized by an ovate body, a prominent caruncle with three lobes, dendritic branchiae, and double dorsal cirri. Twenty-two species belonging to *Notopygos* have been described, mostly from the Indo-Pacific region. In America, few species are frequently recorded: *Notopygos crinita* Grube, 1855 from St. Helena Island (Atlantic) and *Notopygos ornata* Grube and Ørsted in Grube 1857 from Costa Rica (Pacific). *Notopygos crinita* is a widely distributed species in the Western Atlantic with additional reports in the Mediterranean Sea (as a questionable alien species) and in the Pacific Ocean. However, only the genus features have been considered, consequently some records could be misidentifications. During a revision of materials from collections and the barcode project, ‘Mexican Barcode of Life, MEXBOL’, we found specimens of *Notopygos megalops* and an undescribed species from reef zones in the Caribbean; the former had been considered a junior synonym of *Notopygos crinita*. Herein, *Notopygos megalops* is reestablished and *Notopygos caribea* **sp. n.** is described. A morphological and DNA barcode approach was used to explain the records of *Notopygos ornata* in the Atlantic and to show the differences with the new species, since both species share features such as complex pigmentation patterns, and circular projections in the median lobe of the caruncle.

## Introduction

Polychaetes belonging to the family Amphinomidae are commonly known as fireworms (Hutchings 2000). However, members of *Notopygos* Grube, 1855 do not produce the burning sensation and can be manipulated without negative consequences (Kudenov 1980). Amphinomids are usually abundant in coral reefs and rocky areas; there are also some deep-water genera. *Notopygos* species are less common and some species have been collected in open waters at depths exceeding 200 m (Salazar-Vallejo 1997).

*Notopygos* was erected by Grube (1855) for the type species *Notopygos crinita* Grube, 1855, from St. Helena Island. In the original description Grube did not mention one of the most important features of the genus: the presence of the double dorsal cirri. Nevertheless, two years later this omission was corrected in the description of *Notopygos ornata* Grube and Ørsted in Grube 1857 from the Pacific coast of Costa Rica. Thus, the genus is characterized by an ovate body, a prominent caruncle with three lobes (one elevated central lobe and two flattened lateral lobes), dendritic branchiae, and double dorsal cirri (Fauchald 1977).

Twenty-two species of the genus have been described, most of them from the Indo-Pacific region. The original descriptions of several species are incomplete, since relevant features such as the beginning of branchiae, position of the anal opening and folds of the caruncle were omitted. Consequently, in some cases it is difficult to delimit and identify the species (Potts 1909). In the last revision of the genus, Horst (1911) pointed out the lack of knowledge about *Notopygos* species and included all the species that had been described. Regrettably, he omitted the illustration of the pigmentation pattern, even though Potts (1909) had emphasized the importance of pigmentation pattern to distinguish species. Thus, the group is still poorly known and the delimitation of some species is problematic. The lack of knowledge is evident in *Notopygos ornata*, which was described in 1857, but some features were not characterized until almost eighty years later when Monro (1933) redescribed the species, including worms of different sizes, and found some variations in juvenile specimens. In addition, he observed that the pigmentation pattern does not deteriorate once the specimen has been preserved. That contribution included other important observations because it considered the variability in 18 specimens.

*Notopygos crinita* is widely distributed in the Western Atlantic. In addition, there are reports of the species in the central Mediterranean where it was considered to be alien (Zenetos et al. 2010) and as a non-established alien species on the Italian coast (Occhipinti-Ambrogi et al. 2011). It was even recorded in the Pacific Ocean (Cocos Island) by Treadwell (1928), but Dean (2004) regarded this record as doubtful. In the Western Atlantic region, only one more *Notopygos* species has been described, *Notopygos megalops* McIntosh, 1885; this was described based on a juvenile specimen from Bermuda and it was differentiated from the other species by differences in the caruncle, the branchiae beginning on chaetiger 6 and form of cirri. Hartman (1959) suggested that this species could be a junior synonym of *Notopygos crinita*, although she did not review the type material of the two species; her statement was supported by Ebbs (1966). Salazar-Vallejo (1997) revised the holotype of *Notopygos megalops* and some additional specimens collected in the Mexican Caribbean and validated the synonymy. Therefore, the only species of the genus recorded in the Greater Caribbean basin is *Notopygos crinita*, with some doubtful records of *Notopygos ornata*.

During a revision of preserved material from museum collections and newly collected material from the barcode project “Mexican Barcode of Life, MEXBOL”, we found an adult form of *Notopygos megalops* and an undescribed species from reef zones in the Caribbean. Herein, *Notopygos megalops* is reestablished and a new species of *Notopygos* is described. In addition, DNA barcoding was used to complement the morphological approach in order to explain the records of *Notopygos ornata* in the Atlantic and to better differentiate the new species, since both have a similar pigmentation pattern.

## Methods

Reviewed materials belong to the following collections: The Natural History Museum, London (BMNH); Reference Collection (ECOSUR) and collection of ethanol-fixed specimens (ECOSUR-OH) of El Colegio de la Frontera Sur, Chetumal; Marine Invertebrate Museum, Rosenstiel School, University of Miami (UMML); and National Museum of Natural History, Smithsonian Institution, Washington (USNM).

Material from ECOSUR-OH was collected by snorkelling in the reef lagoons; worms were removed from coralline rocks and fixed in 96% ethanol. To observe the morphological attributes, we used Shirlastain A and methyl green to bring out details. Specimens were measured to record the width (in the widest part without chaetae), the body length (from prostomium to pygidium), appendage length, caruncle length and width, branchial filaments by branchiae and distal lobes length and width. All of the appendages and caruncle were measured directly under the microscope with a miniscale (0.1 mm divisions). Taxonomical features were illustrated with microphotography using a Cannon Rebel EOS and line drawing to caruncle and pigmentation pattern. Semi-permanent slides of chaetae from chaetigers 1, 3, 10 and 15 (often including some additional) were prepared to describe the chaetal features. Chaetae were measured with a calibrated microscope scale and SEM analyses were performed.

DNA barcoding for *Notopygos ornata* and the new species followed standard protocols (Hajibabaei et al. 2005). The specifications for polychaetes are described in Carrera-Parra and Salazar-Vallejo (2011). Sequence data, electropherograms, trace files, primer details, photographs and collection localities for specimens are available within the project Polychaeta of Mexico II, Barcode of Life Data System BOLD (http://www.barcodinglife.org; Ratnasingham and Hebert 2007).

Sequences were aligned with ClustalW interface MEGA version 5 (Tamura et al. 2011). Anterior and terminal ends were removed. Sequence divergences were calculated with the Kimura two parameter (K2P) distance model (Kimura 1980) as standard model for constructing genetic distance matrices in BOLD. Neighbor-joining (NJ) tree was created as a representation of the divergence pattern between species (Saitou and Nei 1987). The sequence from *Hermodice carunculata* (also in BOLD) was incorporated as outgroup.

## Results

### Family Amphinomidae Savigny in Lamarck 1818. Genus *Notopygos* Grube, 1855

#### Notopygos caribea sp. n.

urn:lsid:zoobank.org:act:6D3A9313-282E-4297-87B3-FDD4E6756370

http://species-id.net/wiki/Notopygos_caribea

Figures 2 , 4c, f

Notopygos crinita Treadwell 1901:194 (*partim, non*Grube 1855).

##### Type-material.

**Figure 1.**
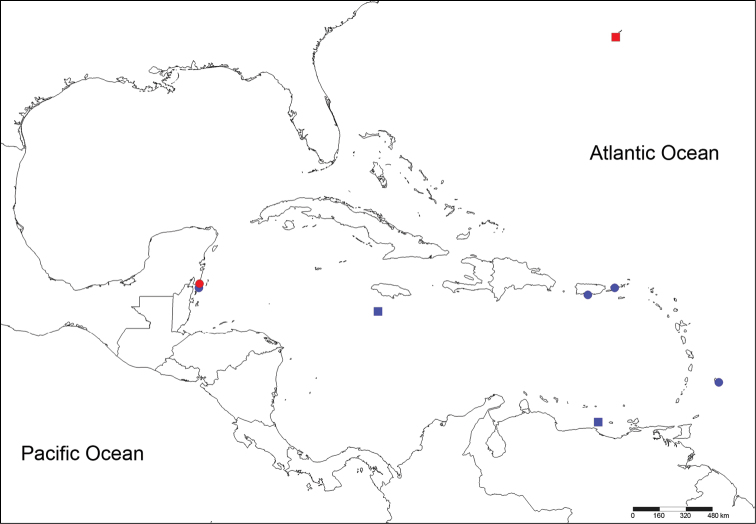
Localities from material reviewed. Circles: *Notopygos caribea* sp. n., squares: *Notopygos megalops*, red: type material, blue: additional material.

Holotype [ECOSUR 0145 (ECOSUR-OH-0213)] Xahuayxhol, Quintana Roo, México, 18°30'30"N, 87°44'02"W, August 2004, 1 m, reef lagoon, coralline rock, Coll. LFCP. Paratypes (3) ECOSUR 0146, 0147, 0148] the same data as for holotype (Fig. 1).

##### Additional material.

[ECOSUR P2641] Xcalak, Quintana Roo, México, sta. 4, 18°16'57"N, 87°49'8"W, in nightlight lift-net, August 2005, Coll. L. Vazquez. [USNM 15921] Sail Rock, Off Saint Thomas, 37 m, coral; [USNM 20296] Pillars of Hercules, English Harbor, Antigua; [USNM 15859] Puerto Rico; [USNM 20273] Barbados (Fig. 1); all as *Notopygos crinita*.

##### Description.

**Figure 2.**
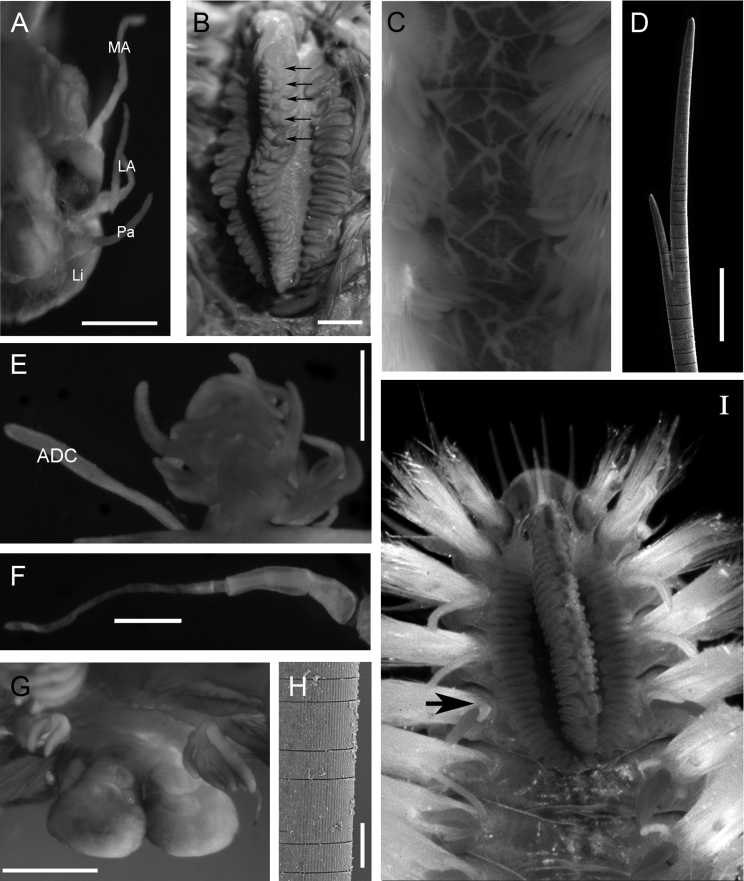
*Notopygos caribea* sp. n. **A** Prostomium, dorsal view **B** Caruncle, arrow showing circular projection in the middle of the caruncle **C** Pigmentation pattern between chaetiger 9–12 **D** Notochaeta from chaetiger 10 **E** Branchia and accessory dorsal cirrus from chaetiger 15 **F** Main dorsal cirrus from chaetiger 15 **G** Distal lobes **H** Chaetal fragmentation **I** Anterior part of live specimen from Guana Island, BVI (photo: Leslie Harris), arrows showing branchiae beginning. Holotype: **A, B, E–G**, paratype: **C, D, H**. **ADC** accessory dorsal cirrus **LA** lateral antennae **Li** lips **MA** median antenna **Pa** palps. Scale bar: **A, B, E-G**= 0.5 mm, **D**= 100 μm.

Holotype (ECOSUR 0145) mature male, complete, with 30 chaetigers, 3 cm total length, 1 cm wide. Body fusiform, orange to light brown, with reddish-brown branchiae. Pigmentation pattern complex, triangular and rhomboid forms covering the dorsum (Figs 2C, 4F). Prostomium semicircular with four eyes in pigmented strip, anterior eyes twice size of posterior ones (Fig. 2A). Median antenna in central position on prostomium, long and slender (1.1 mm long, Figs 2A, 4C); pair of lateral antennae placed on anterior prostomial margin, size similar to median antenna (1 mm long). Lips with lateral palps, shorter than lateral antennae (0.7 mm). Mouth ventral between peristomium and chaetiger 3.

Caruncle oval (2.9 mm long, 1.7 mm wide), with an elevated central lobe with about 20 folds (Fig. 4C). Row of circular projection protruding between each pair of folds in middle of caruncle (Figs 2B, 4C). Lateral lobes flattened with pigmented base and folding edge with 19 and 22 folds (Fig. 4C).

Branchiae from chaetiger 5 (Fig. 2I), present throughout body. Each branchia with main short stem, branching in several filaments of various thicknesses and lengths (Fig. 2E). First branchia with 11 branchial filaments, second branchia with about 20, middle one with about 40, posterior branchiae with 35–45 filaments up to the last chaetiger, where filaments are fewer. The large number of filaments causes a secondary ramification without a defined pattern.

Parapodia biramous, notopodium with double cirri and neuropodium with single ventral cirrus. Notopodial cirri differing; accessory cirrus (= branchial cirrus) simple with similar length along body (1.0–1.2 mm); dorsal cirrus with short, thick cirrophore (0.5 mm) in first chaetigers, subsequent ones with long slender cirrophore (1 mm) and cirrostyle (1.5 mm, Fig. 2F). Ventral cirri similar along body, cirrophore short (0.2 mm) and cirrostyle long (1 mm), decreasing towards last chaetigers.

Noto- and neurochaetae all asymmetrically furcated, slender (<0.03 mm), ratio of difference between short and long tines varies from three to four times (Fig. 2D). First chaetigers with some notochaetae with extra long tines, 10 to 30 times longer than short tines. Both neuro- and notochaetae include short and long types; shorter chaetae on exterior edge of chaetal lobe. Chaetae of first chaetigers with serrated margin. Some chaetae with an external “hard cover” that easily breaks up, giving the impression of being articulated (Fig. 2H).

Anus dorsal, on chaetiger 23. Posterior end margin with pair of distal lobes (0.5 mm long, 0.5 mm wide in the widest part, Fig. 2G).

Gametes: Gametes are located in the coelom. Oocytes are 40–57 μm in diameter (mean: 28.7±8.3 μm, n=20, one paratype female). Spermatozoids have a spherical head (~ 3 μm), ect-aquasperm type, aggregated in a mass (holotype).

Variation: Material examined varied in total length from 1.0 to 2.1 cm, in width from 0.3 to 0.5 cm, chaetigers from 23 to 26, and varies in the following features. Prostomium: median antenna similar length to lateral ones (from 0.4 mm to 1 mm), palps shorter (0.3–0.7 mm). In some worms the pigmented strip on the prostomium continues to the buccal lips, around the palps, and even onto the ventral body with a dark region, although in holotype this pigmentation is faded. Caruncle fold number varies from 13 in smallest to 20; in all specimens number of folds between elevated lobe and laterals is very similar (±2). Pigmented circular projections in mid-caruncle faded in some preserved specimens. Number of branchial filaments is size-dependent, smallest specimens having only three to nine, largest specimens having up to 25 filaments in median region. Branchiae from chaetiger 5 in both juvenile and adults.

##### Etymology.

The specific name refers to the distribution area of the species.

##### Distribution.

Greater Caribbean basin in shallow waters, related to coralline areas, particular associations are unknown.

##### Remarks. 

*Notoygos caribea* sp. n. is characterized by the complex pigmentation pattern covering the dorsum with triangular and rhomboid forms (Figs 2C, 4F), and by other features such as branchiae beginning on chaetiger 5, anus on chaetiger 23, and a prominent caruncle with a median keel with a series of highlighted points arranged in a a longitudinal row of circles. The juvenile specimen also shows the coloration pattern and the distinctive caruncle. The coloration pattern on the caruncle of some specimens is faded; however, it is possible to distinguish the serial projections on the mid-caruncle.

The most common species recorded in the Atlantic is *Notopygos crinita*. This species was briefly described by Grube (1855) from St Helena. He did not comment on pigmentation pattern and omitted the presence of the second dorsal cirri; furthermore, chaetae are described only as pale yellow, long and asymmetrically bifurcated. Unfortunately, the holotype of *Notopygos crinita* [ZMB Verm. 3330] in the Zoologisches Museum, Berlin is lost (Hartwich 1993), so new topotypical materials are needed for a complete redescription, but this is beyond the scope of this study. However, the differences in anus position (21 vs. 23 in *Notopygos caribea* sp. n.), size of the second cirri (indistinguishable vs. prominent in *Notopygos caribea* sp. n.), pigmentation pattern (unstated, as not denoting vs. complex in *Notopygos caribea* sp. n.) and caruncle (crenulated vs. well-defined structure in *Notopygos caribea* sp. n.) allowed us to separate the two species.

Kinberg (1910) recorded one specimen from St Helena as *Notopygos crinita*; however, the illustration shows one dorsal cirrus per notopodium and his description confirms this character. In addition, Kinberg stated that the specimen lacks the dorsal anus; thus we consider that this specimen does not belong to *Notopygos*, possibly a juvenile *Chloeia*.

Material from Puerto Rico on corals revised by Treadwell (1901) was identified as *Notopygos crinita*; however, in the description he refers to the row of small dark brown bead-like elevations on the median fold of the caruncle. The mention of this characteristic feature of the caruncle in the Treadwell specimens allows us to assume that the Puerto Rico specimens belong to *Notopygos caribea* sp. n.

In the Indo-Pacific region, eight *Notopygos* species have branchiae beginning on chaetiger 5 (Table 1):

**Table 1.** Comparison of relevant features in some *Notopygos* species. For branchiae start and anus position, the numbers indicate chaetigers.

**Species**	**Branchiae beginning**	**Caruncle**	**Pigmentation pattern**	**Anus location**	**Distribution**
*Notopygos crinita* Grube, 1855	5	Ovoid, crenulated, ornamented with elevated median lobe	–	21+ Ehlers, 1887 (intersegment 21–22)	Atlantic St Helena
*Notopygos ornata* Grube & Ørsted in Grube 1857	4	About 20 folds in the elevated lobe with a row of ovals in the middle. Lateral lobes with pigmented areas (Fig. 4E)	Complex Triangular and rhomboid forms in symmetric pattern, 50% of cover (Figs 4E; 5)	24+ Monro, 1933 (23)	Eastern Pacific
*Notopygos megalops* McIntosh, 1885	6	About 6 folds in the elevated lobe with a row of rectangles in the middle. Narrow lateral lobes (Fig. 3B)+ (folded structure)	Only in the cirrophore	18–19+ (undescribed)	Greater Caribbean
*Notopygos rayneri* (Baird, 1870)	5	Three lobes strongly wrinkled. The central lobe detached.	Complex Dorsum violet with white lines crossing in diverse directions	22	Indo-Pacific
*Notopygos flavus* Haswell, 1878	5	Elongated and sinuous. Details undescribed.	Lack of pigmentation	undescribed	Indo-Pacific
*Notopygos variabilis* Potts, 1909	5	Three lobes with slack arrangement. Lateral lobes with pigmented areas (Fig. 4A)	Orange spots like chessboard (live), Unpigmented in preserved material.	22–25	Indian Ocean
*Notopygos sibogae* Horst, 1911	5	Lateral lobes with a dark tone and 16–17 folds. Central lobe undescribed	Each segment colorless with an area having triangular shape. Violet band around the notopodium and only secondary cirri violet	23	Indo-Pacific
*Notopygos cirratus* Horst, 1911	5	Lateral lobes with 11 folds without pigmentation. Central lobe undescribed	Each segment has three areas. Grey with a dark band around the base of each notopodium and violet cirrophore	Intersegment 23–24 on a papilla	Indo-Pacific
*Notopygos gigas* Horst, 1911	5	Lateral lobes with 30 folds and pigmented areas. Central lobe undescribed.	Violet or brown in the middle of dorsum with several white lines. Branchiae and two dorsal cirri pigmented.	25	Indo-Pacific
*Notopygos horsti* Monro, 1924	5	18 folds in the elevated lobe with two rows of small circles. Lateral lobes with light pigmented areas (Fig. 4B)	Dorsum marbled with a dark purple pigment, which covers the basal branchiae portion	Intersegment 22–23	Indo-Pacific
*Notopygos andrewsi* Monro, 1924	5	More than 25 folds in the elevated lobe, without pigmented areas (Fig. 4D)	Dorsum with crossed lines and raised longitudinal ridges (Fig. 4G)	24	Indo-Pacific
*Notopygos caribea* sp. n.	5	About 20 folds in the elevated lobe, with a row of circles in the middle. Lateral lobes with pigmented areas (Figs 2B, I, 4C)	Complex Triangular and rhomboid forms, 90% of cover (Figs 2C, I, 4F, 5)	23	Greater Caribbean

+ Modified after original description; in parenthesis, original data.

1) *Notopygos rayneri* (Baird, 1870) from north-eastern Australia also has a complex pigmentation pattern but with white lines crossing in various directions, whereas *Notopygos caribea* sp. n. lacks white lines even in live specimens (Figs 2I, 5). 2) *Notopygos flavus* Haswell, 1878 from northern Australia lacks any pigmentation pattern. 3) *Notopygos variabilis* Potts, 1909 from the Maldives differs from *Notopygos caribea* sp. n. mainly in caruncle shape (Fig. 4A) and pigmentation pattern of live specimens, which is like a chessboard with orange spots (original description lacking illustration of pigmentation pattern). 4) *Notopygos sibogae* Horst, 1911 from Indonesia differs in that the pigmentation pattern includes a violet band around the notopodium and violet secondary cirri (original description lacking illustration of pigmentation pattern). 5) *Notopygos cirratus* Horst, 1911 from the Philippines differs in the pigmentation pattern grey with a dark band around the base of each notopodium and violet cirrophore, and in the intersegmentary location of the anus above an elevation. 6) *Notopygos gigas* Horst, 1911 from the south coast of Timor differs in the dissimilar anus location and branchiae shape, being three large stems in *Notopygos gigas*, while in *Notopygos caribea* sp. n. the branchial ramification lacks a defined pattern. 7) *Notopygos horsti* Monro, 1924 from northern Australia differs from *Notopygos caribea* sp. n. by having two series of circular projections on the caruncle median lobe (Fig. 4B) instead of only one (Fig. 4C) and by the intersegmental anus position. 8) *Notopygos andrewsi* Monro, 1924 from Christmas Island has a pigmentation pattern with crossed lines and raised longitudinal ridges and caruncle without pigmentation (Fig. 4D, G), different from *Notopygos caribea* sp. n.

In addition, Horst (1911) suggested that *Notopygos gardineri* Potts, 1909 (Amirante Islands) and *Notopygos labiatus* McIntosh, 1885 (Philippine Islands) have the branchiae beginning on chaetiger 5. In Table 1, these species were omitted because the original descriptions do not provide this information. Therefore, to confirm this assertion it will be necessary to re-examine the types as part of a review the genus, with additional material from the Indo-Pacific region.

#### Notopygos megalops

McIntosh, 1885 reinstated

http://species-id.net/wiki/Notopygos_megalops

Figure 3

**Figure 3.**
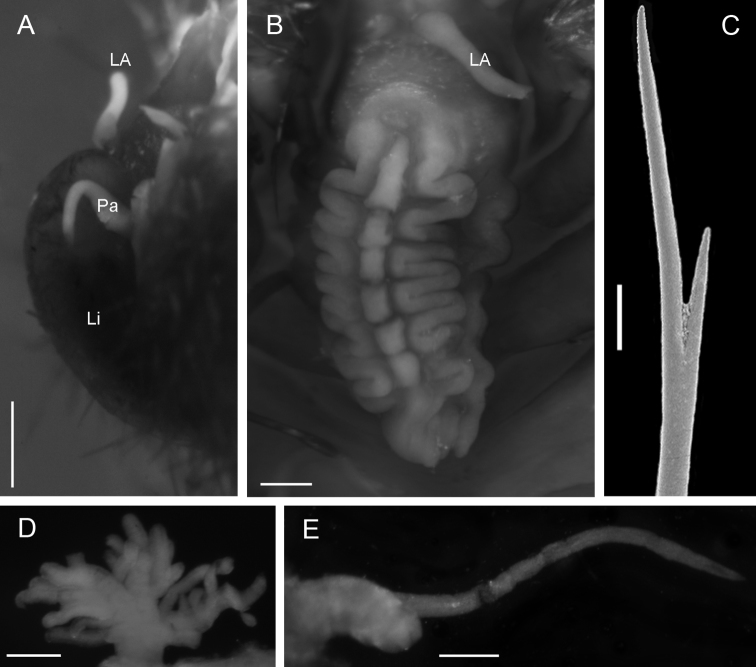
*Notopygos megalops* McIntosh, 1885. **A** Prostomium **B** Caruncle **C** Notochaeta from chaetiger 15 **D** Branchia chaetiger 10 **E** Main dorsal cirrus from chaetiger 15. **LA** lateral antennae **Li** lips **Pa** palps. Scale bar: **A, B, D, E**= 0.5 mm, **C**= 100 μm.

Notopygos megalops McIntosh, 1885: 17–19. Pl. 1, fig. 1, Pl. 2a, fig. 3, 4.
Notopygos crinita Salazar-Vallejo 1997: 384–385 (*partim, non*Grube 1855).

##### Type material.

Holotype (juvenile) [BMNH 1885.12.1.12] Bermuda, 32°07' N, 65°04'W, Sta. 36, “Challenger”, April 1873, 55 m. Broken into two parts, damaged (Fig. 1).

##### Additional material.

[UMML 22.909] Near Jamaica, 17°27'N; 78°10'W, Sta. 1256, “R/V Pillsbury”, July 1970, 590 m. [UMML 22.903] Venezuela, 10°57'N, 66°18'W, Sta. 739, “R/V Pillsbury”, July 1968, 257m, juvenile (Fig. 1).

##### Description, 

Adult specimen (UMML 22.909) complete with 23 chaetigers, damaged, gut exposed, broken, posterior end in poor condition; body fusiform; 3 cm total length, 1 cm wide in the widest part. Prostomium semicircular without pigmented areas, four eyes. Median antenna lost, in central position of prostomium, lateral antennae and palps of similar length (0.7 mm). With stain, ventral surface of buccal lips conserved the stain displaying a glandular zone (Fig. 3A). Mouth placed ventrally in chaetiger 3.

Caruncle oval (1.9 mm length, 0.8 mm wide), elevated lobe with about seven folds in the middle, a rectangular projection between each fold pair. Lateral lobes narrow, with a slightly folded edge, without pigmentation (Fig. 3B).

Branchiae from chaetiger 6, present throughout body. Each branchia as a tuft of slender branchial filaments. First branchiae with a main stem, with seven branchial filaments; in median chaetigers with a main short stem, branching in four stems with five to seven branchial filaments each (Fig. 3D).

Parapodia biramous, notopodium with double cirri and neuropodium with single ventral cirrus. Accessory cirrus simple, long (2 mm); main cirrus with robust short cirrophore (0.5 mm length) and slender and long cirrostyle (1.5–2.0 mm) in all chaetigers (Fig. 3E). Ventral cirri of similar length (1 mm) along the body, last one smallest (0.7 mm).

Chaetae in noto- and neuropodia of two sizes, short and long. All neurochaetae slender (<0.04 mm wide), long notochaetae (Fig. 3C) twice as thick as short notochaetae. All chaetae asymmetrical furcates; ratio of difference between small and large tines is similar in all chaetae, varying from three to four times.

Anus dorsal in the intersegment 18–19. Posterior end margin with pair of short distal lobes.

Gametes: Unknown.

**Figure 4.**
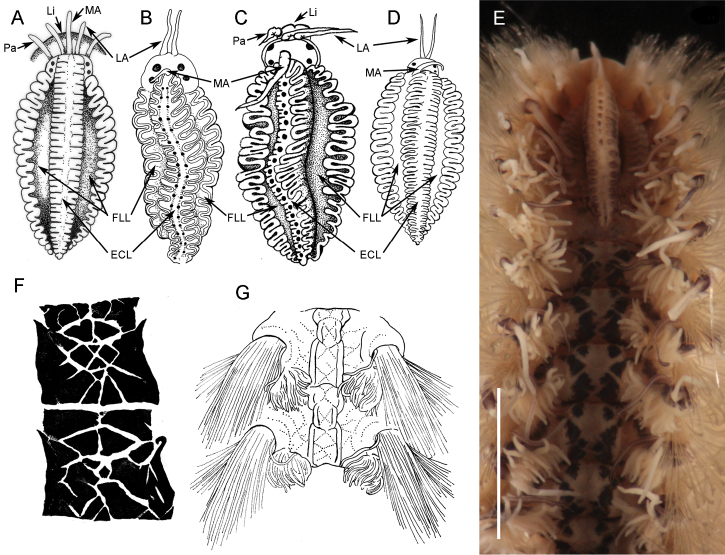
Caruncles and pigmentation pattern of some *Notopygos* species. **A** Caruncula of *Notopygos variabilis* **B** Caruncula of *Notopygos horsti* **C** Caruncula of *Notopygos caribea* sp. n. **D** Caruncula of *Notopygos andrewsi* **E** Caruncula and pigmentation pattern of *Notopygos ornata* from Mexican Pacific **F** Pigmentation pattern of *Notopygos caribea* sp. n. between chaetigers 6–7**G** Pigmentation pattern of *Notopygos andrewsi* anterior chaetigers. Redrawn from original descriptions: **A,B,D,G**. **ECL** elevated central lobe **FLL** flattened lateral lobe **LA** lateral antennae **Li** lips **MA** median antenna **Pa** palps.Scale bar: 3.5 mm.

##### Distribution.

Greater Caribbean, 55 to 590 m.

##### Remarks.

*Notopygos megalops* is characterized by branchiae beginning on chaetiger 6, with four main stems on median chaetigers; anus dorsal in the intersegment 18–19 and a short caruncle in comparison with other *Notopygos* species. The caruncle has a wide median lobe with seven wide folds and narrow lateral lobes. McIntosh (1885) indicated that only the cirrophore has a buff pigmentation; the material reviewed, including the holotype, lacks pigmentation, so we cannot corroborate this statement. McIntosh emphasized the serrations of neurochaetae, and the material examined enabled us to clarify that serrations are present in both noto- and neurochaetae on the first chaetigers.

The main attributes that permit us to associate the juvenile described in the original description with the adult forms are the caruncle, the position of the first branchiae, branchial branching, largest notochaeta and the stout cirrophore. McIntosh (1885: 18) described the caruncle as a “usual folded structure, a little more lax than in *Chloeia*”. In *Chloeia* the lateral lobes usually are narrower than in *Notopygos*, which generally are large and flattened. The caruncle in *Notopygos megalops* has narrow lateral lobes, and relaxed folds in the median lobe (Fig. 3B).

Hartman (1959) suggest that *Notopygos megalops* is a synonym of *Notopygos crinita*. Ebbs (1966) pointed out the differentiation in the details of branchial branching discussed by MacIntosh (1855) to differentiate the two species; however, he considered that the differences in chaetae were only minor variations as in other amphinomids. Thus, he supported the statement by Hartman. Salazar-Vallejo (1997), despite having reviewed the holotype of *Notopygos megalops*, followed Hartman’s opinion and regarded it as a junior synonym of *Notopygos crinita*.

Only two species of the genus have branchiae beginning on chaetiger 6: *Notopygos megalops* and *Notopygos hispidus* Potts, 1909 from the Seychelles. The latter has a complex pigmentation pattern, and a well-developed caruncle with expanded lateral lobes, with about 20 folds and continuous projection between folds in the elevated lobe. In addition, the anus is on chaetiger 21. We consider that there are sufficient features to distinguish *Notopygos megalops* from the other *Notopygos* species; thus, we regard it as a valid species.

The caruncle in juvenile specimens is not completely developed; however, the branchiae beginning on chaetiger 6 and the large size of the notochaetae permit species identification.

### Amphiamerican species and DNA taxonomy: barcoding species delimitation

Amphinomid species have been reported from both the Atlantic and Pacific oceans of America; however, some misidentifications could have occurred because there is a lack of complete species descriptions, illustrated guides and identification keys. This is the case for *Notopygos ornata*: Fauchald (1977) indicated that this species occurs in warm waters in the western Atlantic and eastern Pacific. The similarity between *Notopygos ornata* and *Notopygos caribea* sp. n. involves the caruncle features and pigmentation pattern; in both species, the caruncle has row of circular forms in the medial keel (Fig. 4C, E) and a complex pigmentation pattern, which differs in the percentage cover and form. In addition, the beginning of the branchiae and location of the anus are other morphological differences that have not been considered before.

**Figure 5.**
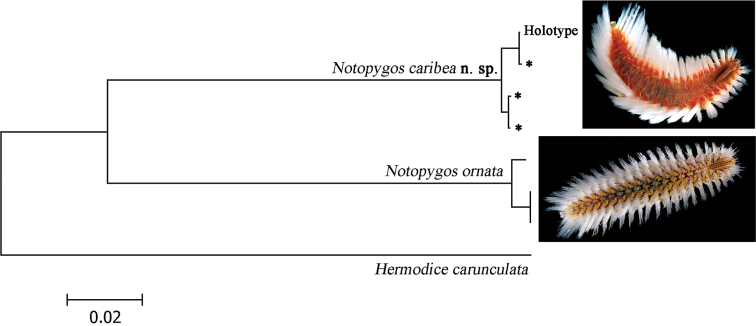
Neighbor-joining tree of COI sequences of two *Notopygos* species (K2P). * sequences from topotype specimens (photos: Leslie Harris).

Eight nucleotide sequences between 618–690 bp of the section of COI gene were obtained to calculate genetic divergence (4: *Notopygos caribea* sp. n., 3: *Notopygos ornata* and 1: *Hermodice carunculata*). The K2P distance between *Notopygos caribea* sp. n. and *Notopygos ornata* shows a genetic divergence around 11% (Fig. 5). Intraspecific genetic divergence in *Notopygos caribea* sp. n. was around 0.8% and for *Notopygos ornata* was 0.7%. Previous studies considered that sequences divergences among related polychaete species average from 8.4% to 21% (Jones et al. 2008, Vrijenhoek et al. 2009, Carrera-Parra and Salazar-Vallejo 2011). This result supports our morphological data in considering the two as different species: *Notopygos ornata* with a distribution restricted to the Tropical Eastern Pacific and *Notopygos caribea* sp. n. with a distribution throughout the Greater Caribbean basin.

Recently, a study of populations of *Eurythoe complanata* from the Caribbean, South America and Eastern Pacific has shown high levels of genetic divergence indicating three cryptic species, but the morphological evidence only recognizes one (Barroso et al. 2010). In another amphinomid genus, *Hermodice*,small differences in the general shape of the caruncle lack taxonomical relevance at species level (Yáñez-Rivera and Salazar-Vallejo 2011). However, in *Notopygos* there are enough morphological differences in the caruncle, such as lobe structure, presence/absence of circular projections, and pigmentation pattern, to distinguish species. In addition, the features of the caruncle combined with other morphological features, such as chaetae, branchial arrangement, and anus location, strongly support recognition of *Notopygos* species. The presence/absence of serrated chaetae as Potts (1909) discussed is not a relevant feature, since in all reviewed specimens this kind of chaetae is present in the first segments. One chaetal feature that must be evaluated in these amphinomids is the external cover along all chaetae; sometimes, broken at the tip of the chaetae, it looks like a small cap, as was noted and illustrated by Ebbs (1966). In addition, the fragmentation of this external cover at regular distances gives the impression of small subdistal teeth or serrations along the chaetae. Thus, morphological features should be reevaluated; all *Notopygos* species should be reviewed on the basis of type materials to obtain complete descriptions, and thereby to explain their taxonomic status and phylogenetic relationships within the genus.

The COI genetic divergence between *Notopygos caribea* sp. n. and *Notopygos ornata* is smaller than in another trans-isthmian amphinomids; in *Eurythoe*, the genetic divergence between Pacific and Atlantic clades was 22%; however, the divergence between the two Atlantic species was 10% (Barroso et al. 2010).

The morphological features and the sequence divergence are congruent; both show sufficient differences to distinguish the *Notopygos* species. Other polychaetes, such as in *Eunice* species, show a similar divergence value (12.9%), which has been supported by morphological features (Carrera-Parra and Salazar-Vallejo 2011). The new species described here shows how DNA barcoding can be fully integrated with the morphological approach to recognize species (Padial and de la Riva 2007) through an integrative taxonomy with different kinds of characters to delimit, discover and identify natural species (Will et al. 2005).

## Supplementary Material

XML Treatment for Notopygos caribea

XML Treatment for Notopygos megalops
